# LncRNA MACC1-AS1 associates with DDX5 to modulate *MACC1* transcription in breast cancer cells

**DOI:** 10.1016/j.isci.2023.107642

**Published:** 2023-08-15

**Authors:** Guiyu Zheng, Yanmei Zhu, Liqun Xu, Shaoying Chen, Xiaona Zhang, Wei Li, Weibin Chen, Yanchun Zhou, Wei Gu

**Affiliations:** 1 Department of Pathophysiology, Key Immunopathology Laboratory of Guangdong Province, Shantou University Medical College, Shantou, Guangdong Province 515041, China

**Keywords:** Molecular mechanism of gene regulation, Molecular interaction, Cancer

## Abstract

*MACC1* is a master oncogene involved in multiple aspects of cancer metastasis in a broad variety of tumors. However, the molecular mechanism by which *MACC1* transcription is regulated remains unclear. Here, we show that in breast cancer cells, lncRNA MACC1-AS1 serves as a *cis*-factor to up-regulate *MACC1* transcription and this regulation increases the cell proliferation potential. Mechanistically, MACC1-AS1 forms a complex with DEAD-Box helicase 5 (DDX5) and simultaneously interacts with the distal region of the *MACC1* promoter. The interaction allows its associated DDX5 to spatially contact the *MACC1* core promoter and shift from MACC1-AS1 to the core promoter. Moreover, binding of DDX5 to the core promoter results in local recruitment of the transcription factor SP-1, thus enhancing *MACC1* transcription. Our findings reveal a molecular mechanism by which MACC1-AS1 *cis*-regulates *MACC1* transcription by interacting with the distal promoter region and delivering DDX5 to the core-promoter of the gene.

## Highlights

• MACC1-AS1 complexes with DDX5 and meantime interacts with the MACC1 distal promoter
• MACC1-AS1 mediates its associated DDX5 to bind to the core promoter of the MACC1 gene
• Binding of DDX5 to the core promoter recruits SP-1 and activates MACC1 transcription

## Graphical abstract

**Figure:**
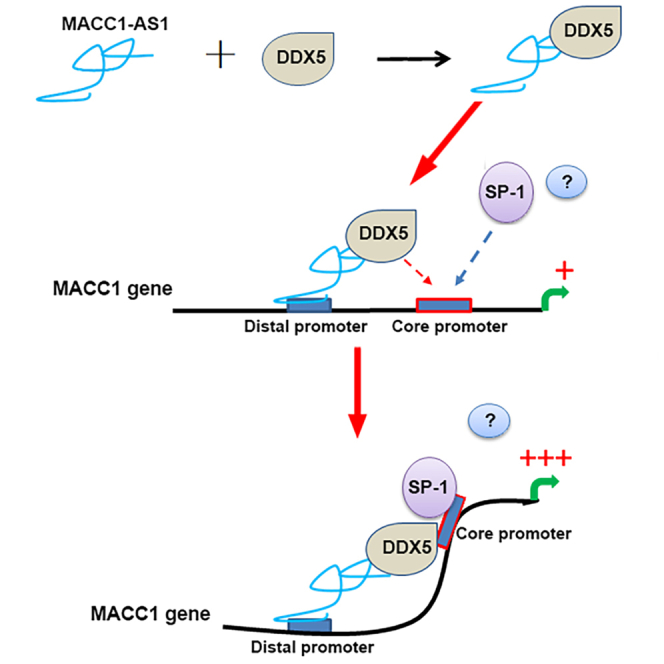

## Introduction

Breast cancer is the most frequently diagnosed cancer and is the second cause for cancer-related death in women.^1^ Since metastasis is responsible for the high mortality among breast cancer patients, the metastatic process is directly related to patient survival and requires the identification of targets for treatment. *MACC1*, originally known as a metastasis-associated in colon cancer 1 gene, is now considered as a master oncogene that promotes proliferation, metastasis, and chemotherapy resistance in a variety of tumors including breast cancer.^2^^,^^3^^,^^4^^,^^5^^,^^6^ A major function of *MACC1* is to regulate the transcription of the *MET* gene via binding to the *EMT* gene promoter, resulting in epithelial-mesenchymal transition (EMT) and metastasis.^7^^,^^8^ *MACC1* is located on human chromosome 7 (7p21.1) and contains seven exons and six introns.^9^ Studies indicate that in human colorectal cancer cells, the proximal flanking region of *MACC1* (−426 nt to −18 nt) is the core promoter that harbors functional elements for binding of transcriptional factors and is responsible for basal transcription of MACC1 mRNA.^10^ However, the potential mechanism by which *MACC1* gene transcription is regulated during tumorigenesis remains unclear.

Long noncoding RNAs (lncRNAs) are defined as a group of abundant transcripts longer than 200 nucleotides and have no protein-coding ability.^11^^,^^12^ To date, numerous lncRNAs have been reported to perform roles in cancer-related gene expression and shown to be responsible for cancer progression.^13^^,^^14^ Depending on their subcellular localization, lncRNAs impact gene regulation through a variety of pathways at both transcriptional and posttranscriptional levels.^15^^,^^16^ LncRNAs that exhibit distinct nuclear localization patterns often function as important modulators to regulate local gene expression either by chromatin reorganization or recruiting transcription factors to the gene promoter,^17^^,^^18^ whereas lncRNAs that are exported to the cytoplasm play regulatory roles to post-transcriptionally mediate mRNA translation or to act as ceRNAs for particular miRNAs.^19^^,^^20^

MACC1-AS1 is an antisense RNA of the sixth intron of the *MACC1* gene. Recent studies have reported that in gastric cancer cells, MACC1-AS1 promotes metabolic plasticity via AMPK/Lin28-mediated stability of MACC1 mRNA and induces fatty acid oxidation-dependent chemoresistance through regulating miR-145-5p activity.^21^^,^^22^ In breast cancer cells, we have previously shown that MACC1-AS1 is distributed in both the cytoplasm and nucleus. The cytoplasmically localized MACC1-AS1 coordinately interacts with PTBP1 and multiple miRNAs to modulate the expression of target mRNAs.^23^ In this study, we mainly used MDA-MB-231 and BT-549 breast cancer cell lines to reveal the function of nuclear localized MACC1-AS1 in upregulation of *MACC1* transcription. We identified that in the nucleus, MACC1-AS1 forms a complex with DEAD-Box helicase 5 (DDX5) to interact with the distal region (−2000 nt to −1500 nt) of the *MACC1* promoter. Interaction with the distal promoter allows MACC1-AS1-associated DDX5 to contact and invade the core promoter of *MACC1*, thus increasing recruitment of transcription factor SP-1 and activating *MACC1* transcription. Our study reveals a molecular mechanism by which association of MACC1-AS1 with DDX5 *cis*-regulates *MACC1* transcription.

## Results

### MACC1-AS1 promotes *MACC1* expression in breast cancer cells

**Figure 1:**
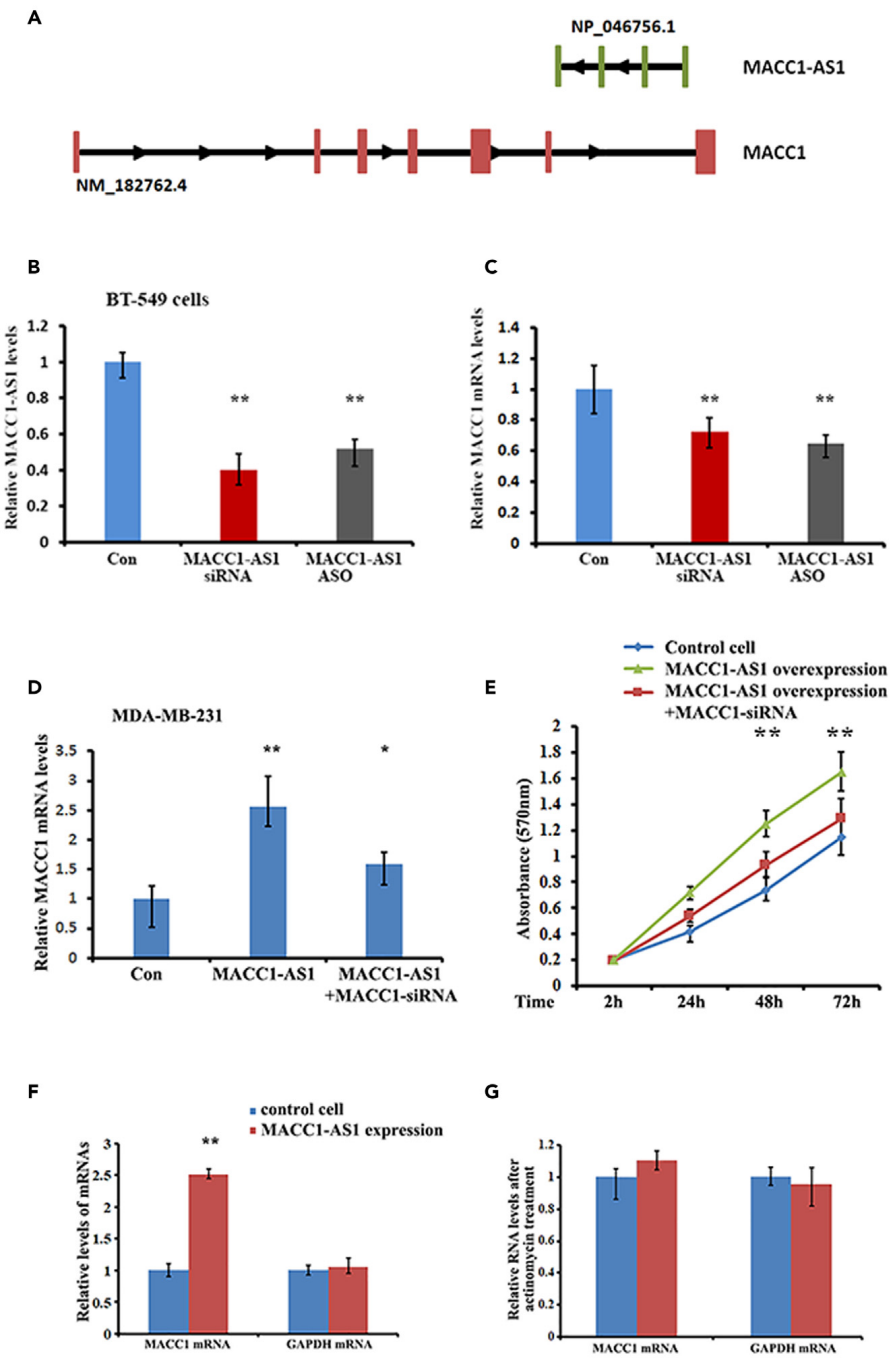
MACC1-AS1 up-regulates MACC1 mRNA expression in breast cancer cells (A) A schematic representation of the genomic structure of lncRNA MACC1-AS1 and the *MACC1* gene. Human MACC1-AS1 is the sixth intron of the opposite strand of the *MACC1* gene on chromosome 7. Exons of the *MACC1* gene and lncRNA MACC1-AS1 are depicted as red or green boxes, respectively, and introns are shown in dark. The arrowhead represents the orientation of transcripts. (B and C) Silencing endogenous MACC1-AS1 in BT-549 cells by siRNA or by ASO (antisense oligonucleotide) decreased *MACC1* mRNA expression. ∗∗p < 0.01. (D and E) MACC1-AS1 induced cell proliferation can be rescued by knocking down MACC1 mRNA expression. ∗∗p < 0.01. (F and G) MDA-MB-231 cells stably expressing MACC1-AS1 were treated with actinomycin D (5μg/ml) for 24 h. Relative levels of MACC1 mRNA were determined by qRT-PCR. Data are normalized to GAPDH mRNA and presented as means ± SD from three independent experiments. ∗∗p < 0.01.

MACC1-AS1, a 639 bp lncRNA, is a cognate antisense RNA of the sixth intron of the *MACC1* gene (Figure 1A). MACC1-AS1 is expressed in various breast cancer cell lines and has been shown to promote cell proliferation and invasion.^23^ Investigation of the relationship between MACC1-AS1 and MACC1 mRNA indicated that MACC1-AS1 can effectively induce *MACC1* expression not only in breast cancer cell lines, but also in mouse xenograft tumor models (Figures S1A and S1B). Knocking down MACC1-AS1 by siRNA or by ASO (antisense oligonucleotide) either in BT-549 cells, which express relatively higher endogenous MACC1-AS1 or in MACC1-AS1-expressing MDA-MB-231 cells, reduced MACC1 mRNA expression (Figures 1B, 1C, and S1C). Moreover, MACC1-AS1 also increased levels of primary MACC1 RNA (Figures S1D and S1E), eliminating the possibility that splicing effect could involve in MACC1-AS1-mediated MACC1 expression. Correlated expression of MACC1-AS1 and MACC1 mRNA was as well shown in human breast tumors by analyzing the GEPIA RNA-seq database (Figure S1F, r = 0.28). Notably, the role of MACC1-AS1 in inducing breast cancer cell proliferation could be rescued by knocking down MACC1 mRNA (Figures 1D and 1E). To analyze whether MACC1-AS1-mediated MACC1 upregulation resulted from mRNA stability, we treated breast cancer cell lines with actinomycin D (5 μg/mL) for 24 h to block *de novo* transcription and measured the endogenous levels of MACC1 mRNA by RT-qPCR. Results showed that cellular levels of MACC1 mRNA were barely affected post-transcriptionally in responding to MACC1-AS1 expression (Figures 1F and 1G). These data suggest the potential of MACC1-AS1 to transcriptionally upregulate *MACC1* expression and this regulation facilitates breast cancer progression.

### MACC1-AS1 interacts with the distal region of the *MACC1* promoter

**Figure 2:**
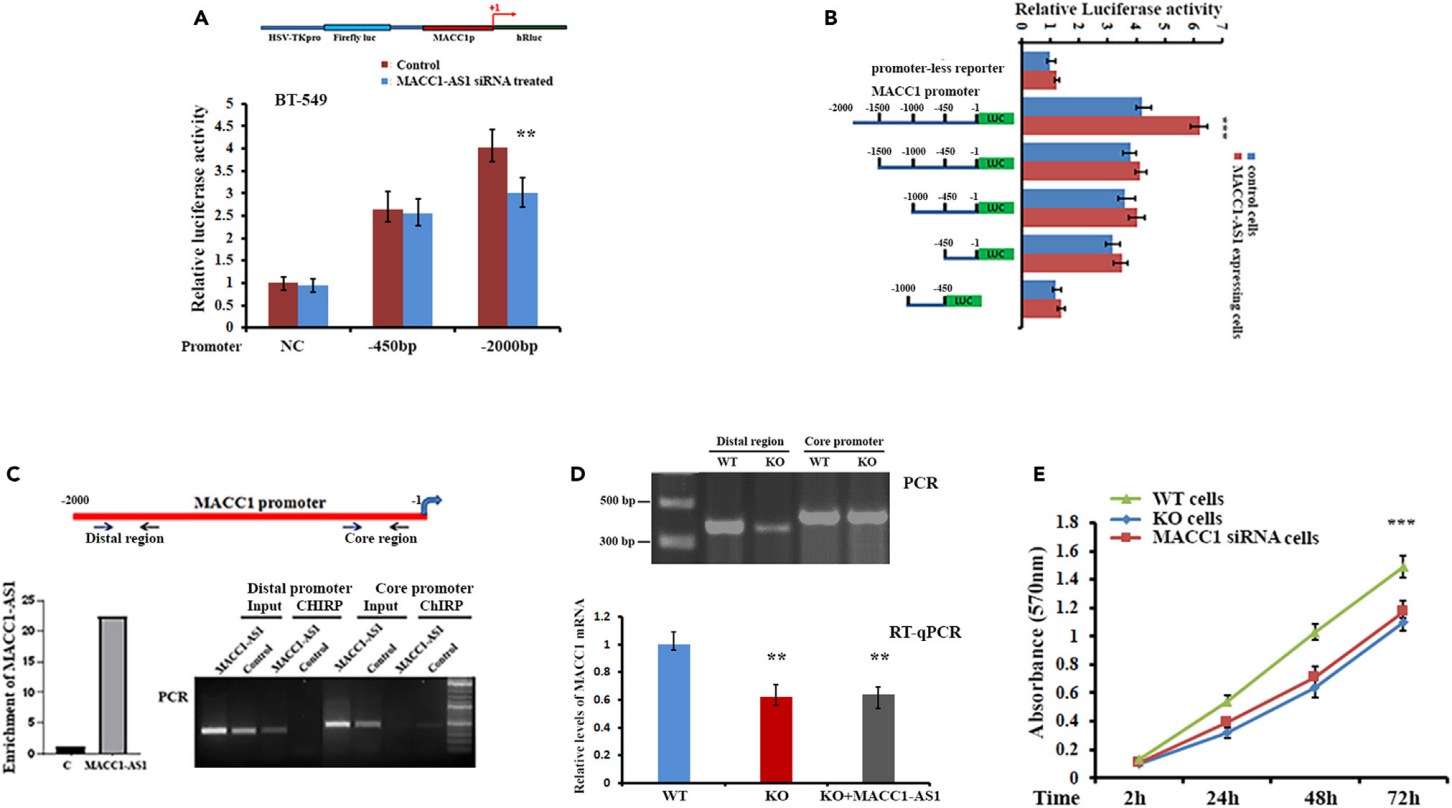
MACC1-AS1 interacts with the distal promoter of the *MACC1* gene (A) Upper: a schematic luciferase reporter in which the −450 bp or −2,000 bp of the *MACC1* promoter (MACC1p) was fused into the 5′-region of the Renilla luciferase gene of the psiCHECK-2 construct. Lower: BT-549 cells with or without MACC1-AS1 siRNA treatment were transfected with the luciferase reporters. Luciferase activities were determined after 36 h transfection using a dual-luciferase assay system. Renilla luciferase activity was normalized to the activity of firefly luciferase. ∗∗p < 0.01. (B) Left: a schematic representation of luciferase reporters driven by different *MACC1* promoters. Right: luciferase activity was determined in MDA-MB-231 cells after transfecting with the individual reporters. ∗∗∗p < 0.001. (C) ChIRP experiments were performed to detect potential interaction of MACC1-AS1 with the *MACC1* promoter in MBA-MB-231 cells. Upper: locations of the two set of primers for amplifying the distal region and the core region of the *MACC1* promoter are indicated with arrows. Lower left: Enrichment of MACC1-AS1 in the ChIRP precipitates were analyzed by RT-qPCR in MACC1-AS1-expressing and control cells. Lower right: PCR indicated that MACC1-AS1 was preferentially interacted with the distal region of the *MACC1* promoter. (D) Upper: PCR and agarose gel electrophoresis showed that in CRISPR edited BT-549 cells (knocking out [KO]), the distal region of the *MACC1* promoter was knocked out. Lower: qRT-PCR indicates the expression of MACC1 mRNA in WT and KO cell. MACC1-AS1 was not able to increase *MACC1* expression when the distal region of the *MACC1* promoter was deleted. ∗∗p < 0.01 as determined by Student’s *t* test. (E) Proliferation assays indicated that either KO of the *MACC1* distal promoter or knocking down MACC1 mRNA decreased cell proliferation. Data are presented as means ± SD from three independent experiments. ∗∗∗p < 0.001.

In colorectal cancer, a core promoter region (−426 to −18) of the *MACC1* gene is essential for transcriptional activity.^10^ We determined whether MACC1-AS1-induced *MACC1* expression is through the core promoter. Transfecting the psiCHECK-2 luciferase reporter driven by the core promoter (−450 to −1) into BT-549 cells displayed no obvious change in luciferase activity when the endogenous MACC1-AS1 was silenced by siRNA (Figure 2A). In comparison, luciferase activity was significantly lower in MACC1-AS1 silenced BT-549 cells when using a reporter driven by a region spans the nucleotides −2,000 to −1 upstream of the *MACC1* gene (MACC1p_2000_) (Figure 2A). Moreover, transfecting the MACC1p_2000_ reporter into MACC1-AS1-expressing MDA-MB-231 cells showed increased luciferase activity (Figure S2A). These results allowed us to hypothesize that MACC1-AS1 modulates *MACC1* transcription through the region in the −2,000 bp outside of the core promoter. We made a series of luciferase constructs driven by different truncated MACC1p_2000_ promoters (Figure 2B, left), ranging from −1,500 nt to +1 (MACC1p_1500_), −1,000 nt to +1 (MACC1p_1000_), −450 nt to +1 (MACC1p_450_), and −1,000 to −500 nt (MACC1p_nC_). Luciferase activity was much lower when the core proximal promoter region was deleted. Interestingly, luciferase activity was also decreased by about 40% in MACC1-AS1-expressing cells following transfection of MACC1p_1500_ reporter lacking the −2,000 to −1,500 nt region (distal region) of the promoter (Figure 2B, right), suggesting that the distal region of the promoter could be the regulatory region mediated by MACC1-AS1. We next performed ChIRP (chromatin isolation by RNA precipitation) assays to investigate the potential interaction between MACC1-AS1 and the *MACC1* promoter. We used MDA-MB-231 and MCF-7 stable cells, in which exogenously expressed MACC1-AS1 was tagged with repeated hairpin motifs (MS2) that allow us to use a recombinant fusion protein (MBP-MCP) to pulldown its associated partners.^24^ We have also used a biotin-labeled antisense oligonucleotide (ASO) that hybridizes to the 5′-end of endogenous MACC1-AS1 in BT-549 cells.^25^ We designed two sets of PCR primers (Figure 2C, upper), one set amplified the distal promoter region (raging from −1,840 to −1,500 nt), and the other set amplified the core promoter region (raging from −450 to −1 nt). ChIRP followed by PCR experiments revealed that either exogenously expressed MACC1-AS1 or endogenous MACC1-AS1 favorably associated with the distal region of the *MACC1* promoter (Figures 2C, lower panel; S2B, and S2C). Recently, CRISPR (clustered regularly interspaced short palindromic repeats)-based genome editing has revolutionized biomedical research, allowing to precisely edit the gene of eukaryotic cells by removal or insertion of genetic information at a desired locus.^26^ To confirm that MACC1-AS1 is the genetic interactant of the distal promoter of the *MACC1* gene, we established a BT-549 cell line in which the *MACC1* distal promoter (−1,840 to −1,527) was knocked out by using CRISPR-Cas9 system (upper panels of Figure 2D). Genetic deletion of the distal promoter region, which was further identified by Sanger sequencing (Figure S2D), reduced *MACC1* expression and this reduction was not able to be rescued by overexpression of MACC1-AS1 (lower panel of Figure 2D). As a results, reduced MACC1 expression in BT-549 cells caused by knocking out of the distal promoter region decreased cell proliferation (Figure 2E). These results indicate that the distal promoter region is responsible for activating *MACC1* transcription by MACC1-AS1.

### Full-length MACC1-AS1 is required for mediating *MACC1* transcription

**Figure 3:**
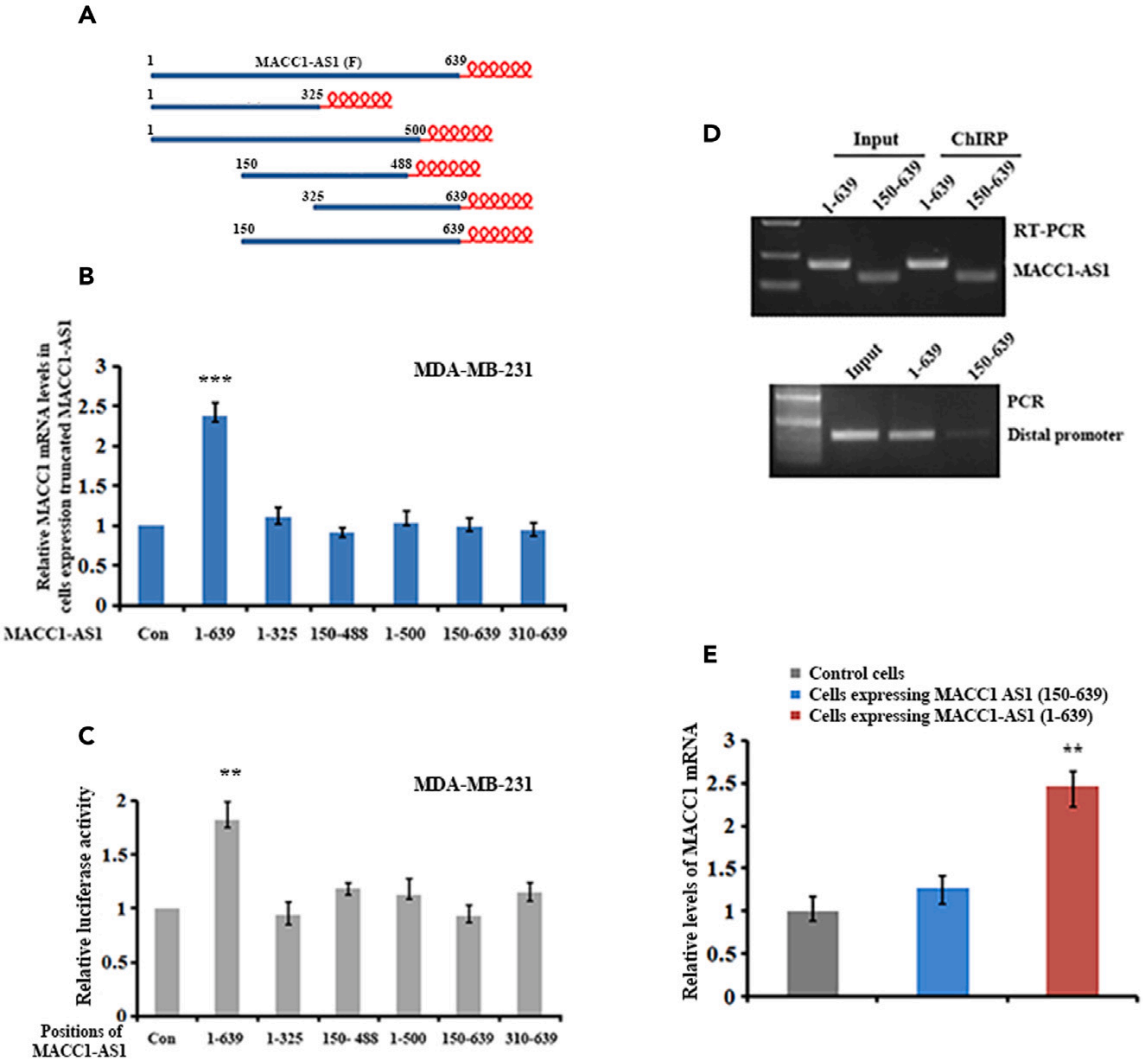
The full-length of MACC1-AS1 is required for activating *MACC1* transcription (A) MACC1-AS1 is dissected into five fragments and are separately tagged with six MS_2_ repeats. The relative position of the truncated MACC1-AS1 is shown. (B) Relative levels of MACC1 mRNA in MDA-MB-231 cells stably expressing full-length and truncated MACC1-AS1 were detected by qRT-PCR, which showed that only the full-length MACC1-AS1 was able to increase MACC1 mRNA expression. ∗∗∗p < 0.001. (C) Luciferase reporter driven by the 2 kb *MACC1* promoter (MACC1p_2000_) was transfected into MDA-MB-231 cells expressing full-length or truncated MACC1-AS1 fragment and cultured for 36 h. Luciferase activity was determined by the dual luciferase reporter system. Activity of Renilla luciferase was normalized to the activity of firefly luciferase. ∗∗p < 0.01, ∗p < 0.05. (D) ChIRP assays indicated that the 5′-truncated MACC1-AS1 (150–639) was not bound to the distal promoter of *MACC1*. Upper panel showed CHIRP precipitated full-length (1–639) and truncated (150–639) MACC1-AS1. Lower panel indicated that the distal promoter of *MACC1* was pulled down by full-length MACC1-AS1. (E) Levels of MACC1 mRNA in MDA-MB-231 cells stably expressing full-length or 5′-truncated MACC1-AS1 (150–639) were analyzed by RT-PCR. MACC1-AS1 truncate was not able to increase MACC1 mRNA expression. ∗∗p < 0.01.

To further investigate the molecular mechanism by which MACC1-AS1 mediates MACC1 transcription, we divided MACC1-AS1 into five segments, each of them was tagged with six MS2 hairpin loop repeats (Figure 3A), and separately cloned them into pCIP2 lentiviral vector. MDA-MB-231 cell lines stably expressing the full-length and truncated MACC1-AS1 lncRNAs were verified by qRT-PCR (Figure S3A). Unlike the full-length MACCl-AS1, all truncated MACC1-AS1 had little effect on increasing *MACC1* expression (Figure 3B). We then transfected the MACC1p_2000_ luciferase reporter into the cells expressing full-length or truncated MACC1-AS1. Luciferase activity was significantly higher in cells expressing full-length MACC1-AS1 than the cells expressing truncated MACC1-AS1 (Figure 3C). Co-transfection of the MACC1p_2000_ luciferase reporter and individual vectors expressing full-length or truncated MACC1-AS1 into breast BT-549 cells gave similar results (Figure S3B), indicating that the full-length MACC1-AS1 was required for facilitating MACC1 mRNA transcription. To determine which region of MACC1-AS1 interacts with the distal promoter, ChIRP experiments were performed in stable cell lines expressing full-length (1–639 nt) or truncated MACC1-AS1 (150–639 nt) (Figure 3D, upper). In contrast to the full-length MACC1-AS1, little amount of the distal promoter was co-precipitated with the truncated MACC1-AS1 (Figure 3D, lower). Moreover, in comparison to cells expressing full-length MACC1-AS1, the cells expressing 5′-truncated MACC1-AS1 has less ability to activate *MACC1* transcription (Figure 3E), suggesting that the 5′-part of MACC1-AS1 interacts with the *MACC1* distal promoter and this interaction promoted *MACC1* transcription.

### Association of MACC1-AS1 with DDX5 enhances *MACC1* transcription

**Figure 4:**
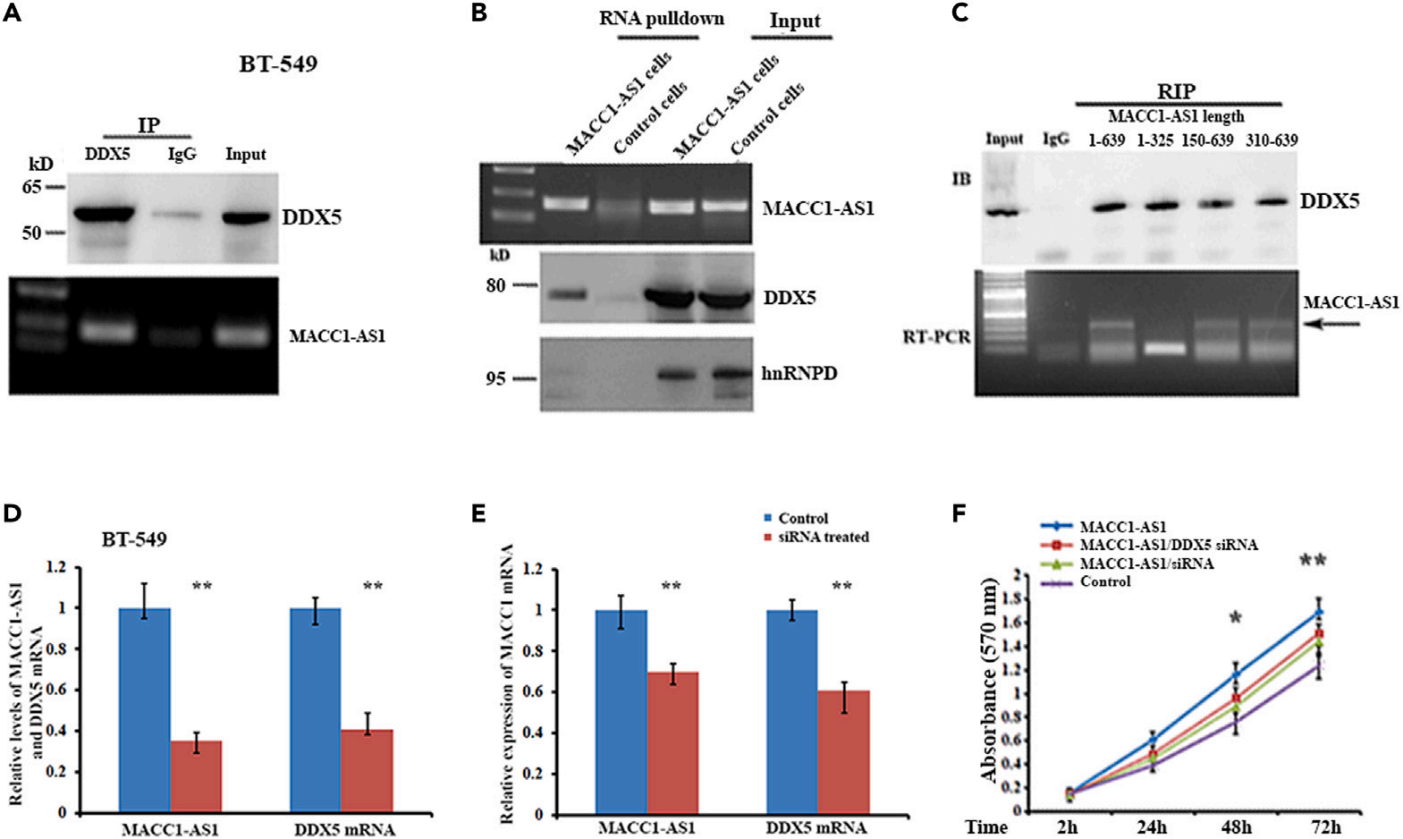
Formation of MACC1-AS1/DDX5 complex is essential for enhancing *MACC1* transcription (A) RNA immunoprecipitation (RIP) to detect the binding of DDX5 to MACC1-AS1. Upper: Western blotting showing immunoprecipitated DDX5. Lower: RT-PCR indicated that MACC1-AS1 was co-precipitated with DDX5. (B) Upper panel: RT-PCR and agarose gel electrophoresis showing precipitated MS2-tagged MACC1-AS1 RNA. Lower two panels: Western blots indicating that DDX5 co-precipitated with MACC1-AS1. hnRNPD was not detected in the precipitates. (C) Upper: RIP assays were performed using DDX5 antibody and the nuclear lysates expressing full-length or truncated MACC1-AS1 (numbers indicate nucleotide sequences of MACC1-AS1). Lower: RT-PCR and agarose gel electrophoresis demonstrated that truncated MACC1-AS1 (1–325) does not bind to DDX5. (D and E) BT-549 cells were treated with MACC1-AS1 siRNA or DDX5 siRNA overnight. Levels of MACC1-AS1 or DDX5 mRNA, as well as MACC1 mRNA were measured by qRT-PCR after siRNA treatment. ∗∗p < 0.01. (F) Proliferation of MDA-MB-231 cells was analyzed. MACC1-AS1 induced cell proliferation can be rescued by either knocking down MACC1-AS1 or DDX5 mRNA. Data are presented as means ± SD from three independent experiments. ∗∗p < 0.01, ∗p < 0.05.

RNA binding-proteins often play roles in mediating the activity of their binding partners.^27^ Using *in vitro* RNA pulldown assays combined with protein sequencing, we identified DDX5, PTBP1, as well as some hnRNPs to be complexed with MACC1-AS1 in breast cancer cells (Figure S4A).^23^ We hypothesized that these proteins, particularly DDX5, could associate with MACC1-AS1 to mediate *MACC1* transcription. To validate the potential interactions between MACC1-AS1 and the precipitated proteins, we prepared cell nuclear extract (Figure S4B) and performed RNA immunoprecipitation (RIP) assays using DDX5 antibody. MACC1-AS1 was directly bound to DDX5 in both BT-549 and MDA-MB-231 breast cancer cells (Figures 4A and S4C). MACC1-AS1 pulldown assays also showed that DDX5 was specifically precipitated with MACC1-AS1, whereas hnRNPD was barely detected in the MACC1-AS1 precipitates (Figure 4B). Further RIP assays using nuclear lysates from cells expressing truncated MACC1-AS1 indicated that DDX5 bound to the 3′-part of MACC1-AS1 (310–639 nt) (Figure 4C). Interestingly, either knocking down MACC1-AS1 or DDX5 mRNA decreased *MACC1* expression (Figures 4D and 4E), as well as cell growth ability (Figure 4F). In addition, luciferase activity was decreased in DDX5 silenced BT-549 cells when using a reporter driven by MACC1p2000 promoter (Figure S4D). These results suggest the significance of MACC1-AS1/DDX5 complex in mediating *MACC1* transcription.

### MACC1-AS1 mediates its associated DDX5 to bind to the core promoter of *MACC1*

**Figure 5:**
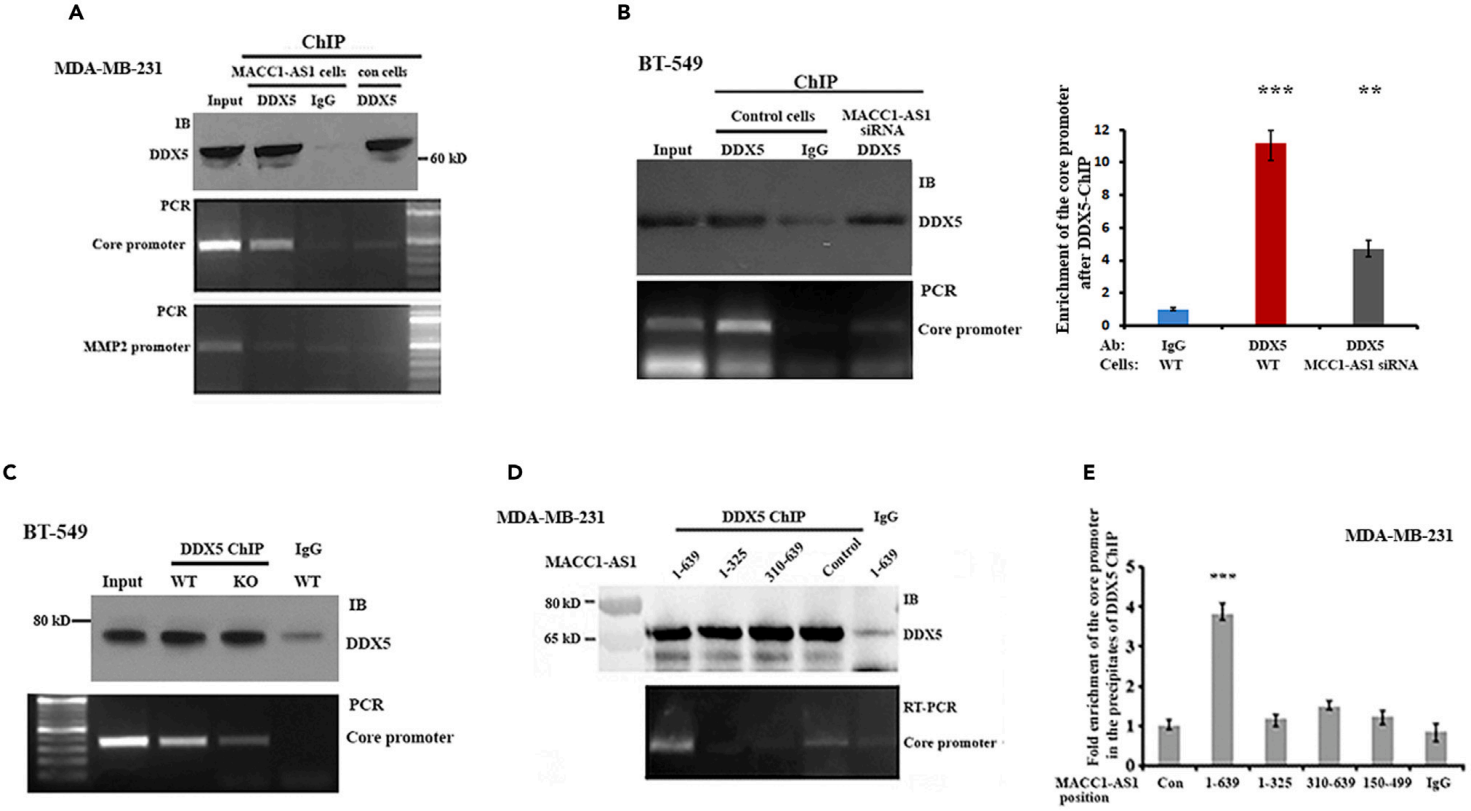
MACC1-AS1 mediates the binding of DDX5 to the *MACC1* core promoter (A) Protein A beads conjugated with DDX5 antibodies were used for ChIP assays in MDA-MB-231 cells with or without MACC1-AS1 expression. Upper penal: Western blots showing immunoprecipitated DDX5. Normal IgG was used as a negative control. Lower two panels: PCR and agarose gel electrophoresis indicated that MACC1-AS1 increased binding of DDX5 to the core promoter of the *MACC1* gene, but not the internal control of the MMP2 promoter. (B) Left upper panel: DDX5 was immunoprecipitated in BT-549 cells in which MACC1-AS1 was silenced by siRNA. Left lower panel: agarose gel electrophoresis showing that MACC1-AS1 knocking down decreased binding ability of DDX5 to the *MACC1* core promoter. Right panel: qPCR confirmed that silencing of MACC1-AS1 significantly reduced the potential of DDX5 to bind to the core promoter of the *MACC1* gene. (C) Upper: DDX5 was immunoprecipitated in BT-549 cells where the endogenous *MACC1* distal promoter was knocked out by CRISPR editing. Lower: PCR and agarose gel electrophoresis showing that knocking out the distal promoter reduced binding of DDX5 to the *MACC1* core promoter. (D) ChIP experiments were performed using DDX5 antibody and cell lysates expressing full-length (1–639) and truncated (1–325 and 310–639) MACC1-AS1 fragments. qPCR indicated that the enrichment of the core promoter in DDX5 precipitates was only appeared in the cells expressing full length of MACC1-AS1. (E) RT-qPCR assays indicated that the enrichment of the core promoter in DDX5 precipitates was higher in the cells expressing full-length MACC1-AS1 than the cells expressing truncated MACC1-AS1.

Next, we investigated the underlying mechanism of MACC1-AS1/DDX5 complex in mediating *MACC1* transcription. DDX5 is a potent RNA- or DNA-binding protein involved in many aspects of gene regulations.^28^^,^^29^ A recent study has demonstrated that DDX5 modulates transcription of the *Myc* gene by refolding its promoter.^30^ We rationalized that interaction of MACC1-AS1 with the *MACC1* distal promoter would eventually allow its associated DDX5 to approach and invade the *MACC1* core promoter. To test this hypothesis, we performed ChIP assays to determine whether MACC1-AS1 could mediate DDX5 binding to the *MACC1* core promoter. By measuring the enrichment of the *MACC1* core promoter or the *MMP2* promoter (internal control) in the DDX5 ChIP precipitates, we observed that the binding potential of DDX5 to the *MACC1* core promoter was largely increased in MDA-MB-231 cells expressing MACC1-AS1 compared to the cells without MACC1-AS1 expression (Figure 5A). No enrichment was seen for a control *MMP2* promoter. Moreover, binding of DDX5 to the core promoter was markedly reduced in BT-549 cells either the endogenous MACC1-AS1 was silenced by siRNA (Figure 5B), or the distal region of the *MACC1* promoter was genetically knocked out (Figure 5C), indicating both MACC1-AS1 and the *MACC1* distal promoter are required for facilitating DDX5 to binds to the core promoter. We next using stable cells expressing truncated MACC1-AS1 to determine which part of MACC1-AS1 would mediate the binding of DDX5 to the core promoter. PCR and agarose gel electrophoresis indicated that in contrast to the full-length MACC1-AS1, truncated MACC1-AS1 either lacking the 5′-region or the 3′-region lost the ability to assist DDX5 binding to the *MACC1* core promoter (Figure 5D). qPCR also detected that the enrichment of the core promoter in DDX5 precipitates was about 3-folds more in the cells expressing full-length MACC1-AS1 than the cells expressing truncated MACC1-AS1 (Figure 5E). These results suggested that interaction of the 5′-region of MACC1-AS1 with the *MACC1* distal promoter would shift its 3′-associated DDX5 to the core promoter of the *MACC1* gene.

### Binding of DDX5 to the *MACC1* core promoter increases the recruitment of SP-1 and activates *MACC1* transcription

**Figure 6:**
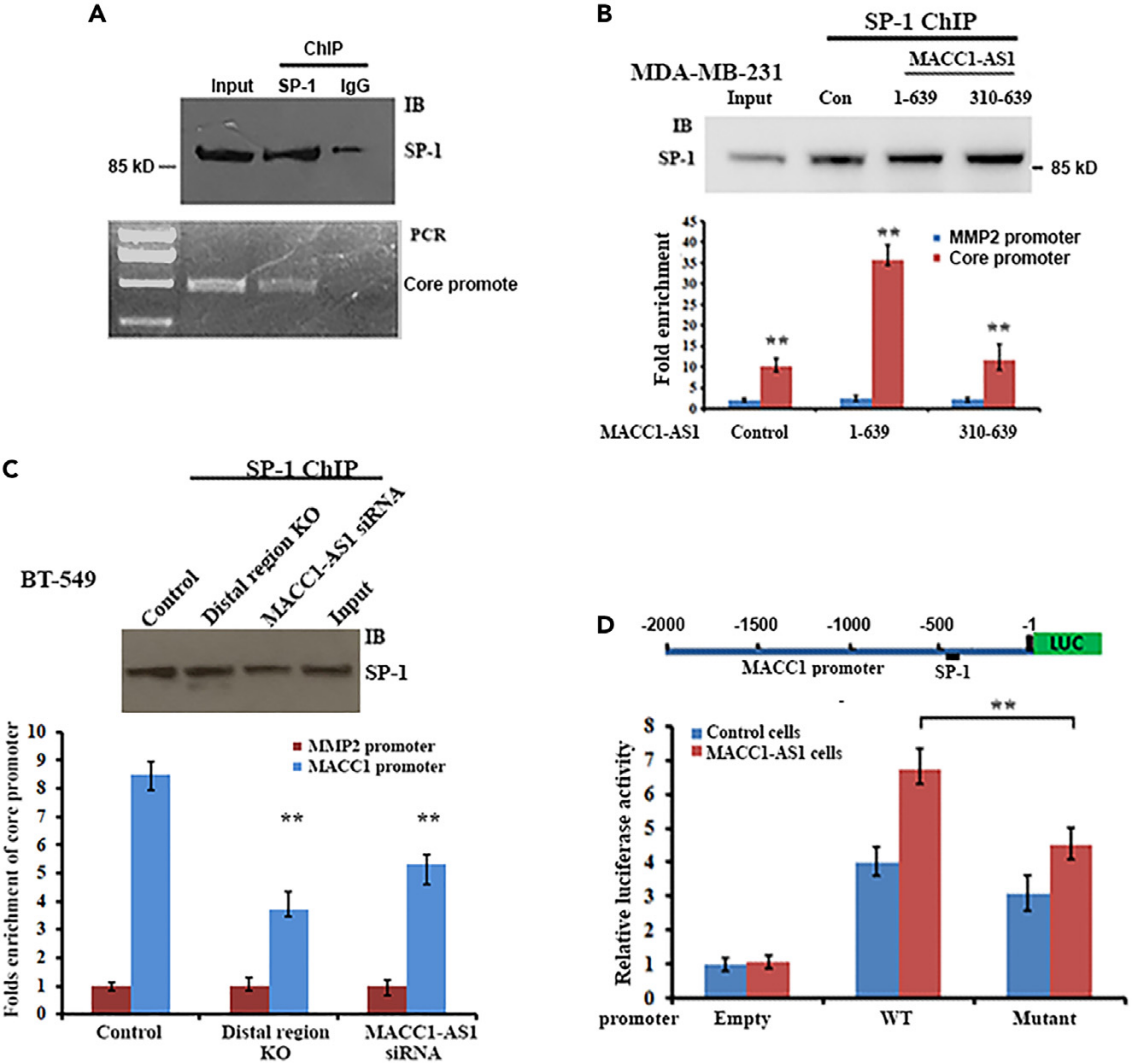
DDX5 elevates recruitment of SP-1 to the *MACC1* core promoter (A) ChIP experiments were performed using SP-1 and normal IgG antibodies. PCR and agarose gel electrophoresis indicated that the core promoter of the *MACC1* gene was enriched in the SP-1 precipitates. (B) ChIP assays were performed to detect the binding ability of SP-1 to the *MACC1* core promoter in cells expressing full-length (1–639) and truncated MACC1-AS1 (150–639). Upper: Western blots show precipitated SP-1. Lower: relative levels of the amplicon corresponding to the core promoter in SP-1 precipitates were analyzed by qPCR. Data are presented as means ± SD from three independent experiments. ∗∗p < 0.01 as determined by Student’s *t* test. (C) Analyzing the binding potential of SP-1 to the *MACC1* core promoter in the MACC1 distal region knockout or MACC1-AS1 silenced BT-549 cells by ChIP assays. The *MMP-2* promoter was used as an internal control. Upper: Western blots indicate the precipitated SP-1 in tested cells. Lower: amplicon corresponding to the core promoter of the MACC1 gene was amplified by qPCR. Relative levels of the core promoter are presented as means ± SD from three independent experiments. ∗∗p < 0.01. (D) Upper: a schematic representation of a luciferase reporter driven by the −2000 bp *MACC1* promoter. Black box indicates the SP-1 binding site. WT: wild-type promoter. Mutant: SP-1 site mutated promoter. Luciferase activity was determined after transfecting the reporter into MDA-MB-231 cells with or without MACC1-AS1 expression. Data are presented from three independent experiments. ∗∗p < 0.01 as determined by Student’s *t* test.

Using RNAInter, an online database (http://www.rna.socirty.org/raid/), we identified that the MACC1 core promoter harbors potential binding sites for transcriptional factors SP-1 and c-Jun (Figure S5A). Luciferase reporter assays indicated that SP-1 performed a major role for the core promoter activity and mutation on the SP-1 binding site apparently decreased basal promoter activity (Figures S5B and S5C). ChIP experiments further demonstrated that SP-1 bound to the core promoter containing the SP-1 binding site (Figure 6A). We hypothesized that the shift of DDX5 from MACC1-AS1 to the core promoter would remodel its structure thus to increase the recruitment of SP-1. By comparing relative enrichments of the core promoter in SP-1 precipitates in cells with or without MACC1-AS1 expression, we observed that SP-1 was more efficiently associated with the core promoter in cells expressing full-length MACC1-AS1 (1–639) than the cells expressing truncated MACC1-AS1 (310–639) that lost the ability to interacts with the distal promoter (Figure 6B). Moreover, binding of SP-1 to the core promoter could be reduced when the MACC1 distal promoter region was knocked out or MACC1-AS1 was silenced by siRNA (Figure 6C). We then constructed a mutant reporter (MACC1p_2000m_), in which the potential SP-1 binding site on the core promoter was mutated (Figure 6D, upper). In comparison to the wild-type promoter, luciferase activity was notably decreased when the mutant promoter was used (Figure 6D, lower), indicating the significance of SP-1 binding for core promoter activity. IP (immunoprecipitation) assays showed that DDX5 and SP-1 were not coprecipitated, eliminating the possibility that the recruitment of SP-1 to the core promoter was resulted from DDX5/SP-1 interaction (Figure S5C). Taking together, these results suggest that MACC1-AS1-mediated binding of DDX5 to the core promoter enhanced recruitment of SP-1 to activate *MACC1* transcription.

### A model of MACC1-AS1/DDX5-mediated *MACC1* gene transcription

**Figure 7:**
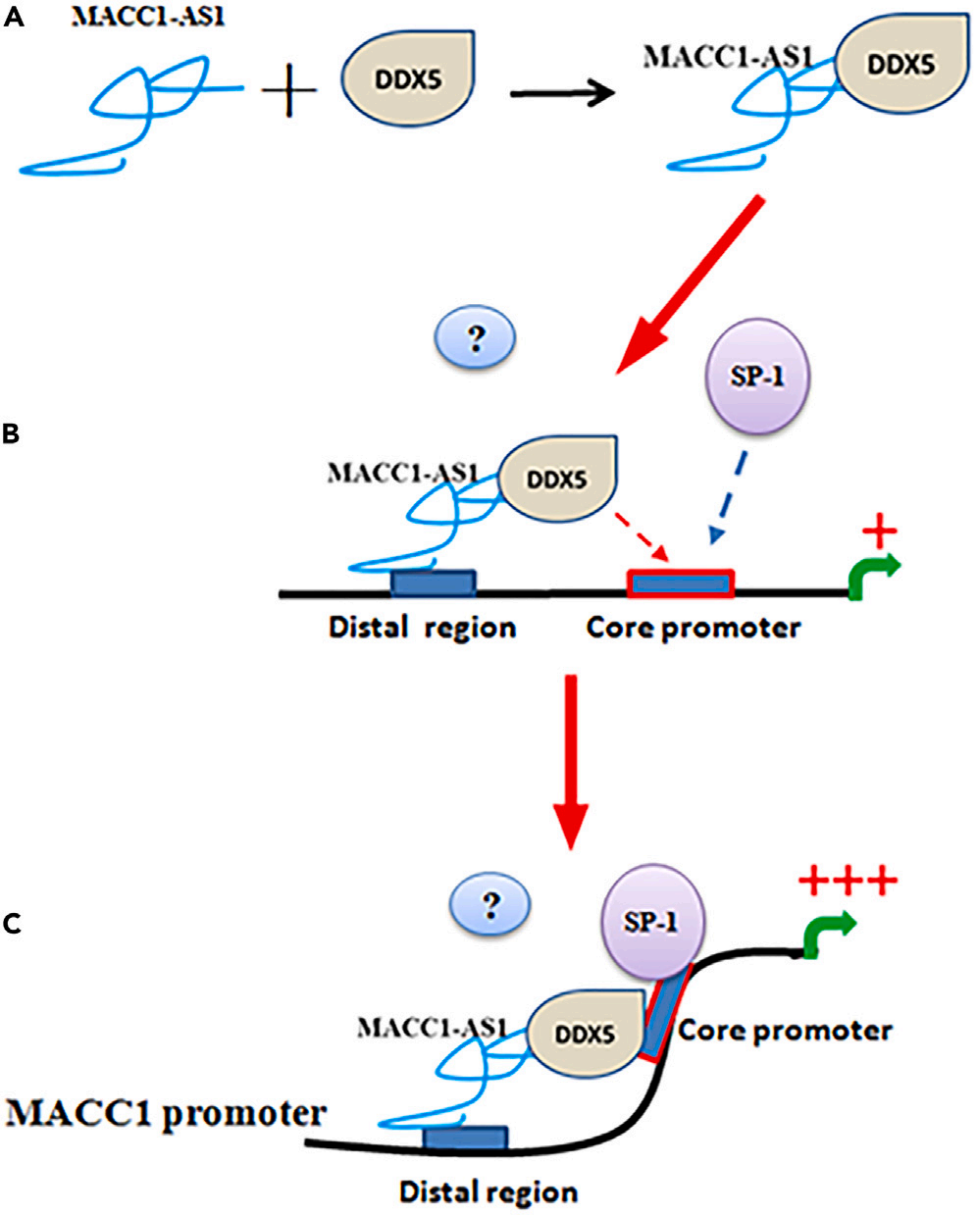
A predicted model for MACC1-AS1/DDX5-mediated *MACC1* gene activation (A) In the nucleus, the 3′-region of MACC1-AS1 forms a complex with DDX5. (B) At the same time, the 5′-region of MACC1-AS1 directly interacts with the distal promoter of the *MACC1* gene. This interaction allows MACC1-AS1-associated DDX5 to close to the *MACC1* core promoter and shift from MACC1-AS1 to the core promoter. (C) DDX5 unwinds the local architecture of the core promoter and recruits transcriptional factor SP-1 to activate transcription.

Based on our findings, we predict a model by which MACC1-AS1 associates with DDX5 to regulate *MACC1* transcription in *cis* (Figure 7). First, the 3′-region of nuclear MACC1-AS1 forms a complex with DDX5, and afterward its 5′-region interacts with the distal region of the *MACC1* promoter. Second, this interaction enables MACC1-AS1-associated DDX5 to come in contact with the *MACC1* core promoter and shift from MACC1-AS1 to the core promoter. Third, binding of DDX5 to the core promoter unwinds the local DNA architecture and recruits transcriptional factor SP-1 to activate *MACC1* transcription. MACC1-AS1 truncates with impaired ability to bind to either DDX5 or to the distal promoter is unable to regulate the transcription of the *MACC1* gene.

## Discussion

*MACC1* was originally shown to be a key regulator of the HGF-MET pathway in colon cancer^8^ and has since been identified as a biomarker in a variety of tumors.^31^ *MACC1* is involved in many aspects of cancer progression including promotion of epithelial-to-mesenchymal transition (EMT), acceleration of cancer metastasis and induction of cell proliferation. In this study, we revealed a novel molecular mechanism by which MACC1-AS1 serves as a *cis*-acting lncRNA to activate transcription of the *MACC1* gene in breast cancer cells. Mechanistically, nuclear localized MACC1-AS1 interacts with a distal region of the *MACC1* promoter. This interaction allows MACC1-AS1-associated DDX5 to contact and invade the core promoter of *MACC1*, thus increasing the recruitment of SP-1 to enhance *MACC1* transcription.

Studies accumulated to date show that lncRNAs are widely expressed in mammalian cells and have key roles in regulating gene expression. Depending on their subcellular localization and their specific interactions with proteins, DNAs or RNAs, lncRNAs can modulate chromatin structure, recruit transcription factors, and mediate the stability and translation of cytoplasmic mRNAs.^16^^,^^32^ LncRNAs, such as MACC1-AS1 that localizes in both cytoplasm and nucleus would perform multiple biological functions. We have previously reported that, in breast cancer cells, MACC1-AS1 functions as a ceRNA (competitive endogenous RNA) for multiple miRNAs to post-transcriptionally regulate the expression of genes targeted by these miRNAs in the cytoplasm.^23^ In this study, we identified a nuclear function of MACC1-AS1 to modulate *MACC1* transcription. While increasing evidence demonstrated the contribution of lncRNAs in oncogenicity, MACC1-AS1 *cis*-regulates *MACC1* transcription provides a key insight toward understanding the molecular mechanism by which lncRNAs execute their oncogenic function.

The functions and mechanisms of lncRNAs to regulate gene transcription are diverse.^32^ One of the major functions of lncRNA is to regulate gene transcription, and this regulation often occurs through association with RNA-binding proteins. LncRNAs could either serve as molecular scaffolds to form functional regulatory complexes via interacting with specific proteins,^33^^,^^34^ or recruit chromatin modifiers to promote gene activation.^35^^,^^36^ Our study indicates that activation of *MACC1* transcription relies on MACC1-AS1 to form an RNA/protein complex with DDX5 via its 3′-region, and then to interact with the *MACC1* distal promoter through its 5′-region. Either knocking out the distal region of the endogenous *MACC1* promoter, or silencing MACC1-AS1/DDX5, decreases the MACC1-AS1-mediated *MACC1* activation. The interaction of MACC1-AS1 with the distal promoter is most likely to allow MACC1-AS1-associated DDX5 to spatially close and invade the core promoter, therefore remodeling the local DNA architecture and making the core promoter more accessible for transcriptional factor SP-1. In this issue, MACC1-AS1 functions as a carrier lncRNA to transport a protein-activator to a gene core promoter through interacting with the gene distal promoter.

DDX5, a member of the DEAD-box RNA helicase family, regulates gene expression in diverse pathways.^29^ DDX5 and its family members not only act as RNA-binding proteins^28^ to regulate mRNA processing and RNA decay, but also co-activate many tumor-related transcription factors in cancer development and progression.^37^^,^^38^^,^^39^ Recently, DDX5 regulates *MYC* gene transcription through interaction with the *MYC* promoter and unfolding the local DNA G4 structure has been reported.^30^ Based on our results, DDX5 functions not only as a lncRNA-binding protein but also a DNA-binding partner to mediate *MACC1* transcription. We propose two roles of DDX5 in the process of *MACC1* activation. First, association of DDX5 with MACC1-AS1 would retain and stabilize MACC1-AS1 in the nucleus and assist MACC1-AS1 to interact with the *MACC1* distal promoter. Second, binding of MACC1-AS1 to the *MACC1* distal promoter allows DDX5 to spatially close and shift to the core promoter, resulting in the unwinding of the local DNA architecture and recruiting transcriptional factor SP-1 to the core promoter.

In conclusion, our study provides a key insight into the lncRNAs in regulation of gene transcription and reveals a molecular mechanism that MACC1-AS1/DDX5 can serve as a *cis*-acting complex to activates *MACC1* transcription.

### Limitations of the study

While our studies show that MACC1-AS1 associates with DDX5 to modulate transcription of the *MACC1* gene, the detailed mechanisms by which MACC1-AS1 interacts with the *MACC1* distal promoter region and DDX5 contacts the core promoter of the *MACC1* gene await further investigation. Addressing these missing links will provide further insights into the molecular mechanism through which MACC1-AS1 *cis*-regulates the *MACC1* transcription.

## STAR★Methods

### Key resources table

**Table:** 

REAGENT or RESOURCE	SOURCE	IDENTIFIER
**Antibodies**		

Anti-DDX5 antibody	BOSTER Biotech (China)	#BM5374
Anti-SP1 antibody	Cell Signaling (USA)	#9389
Anti-MACC1 antibody	ProSci (USA)	#5197
Anti-GAPDH antibody	BOSTER Biotech (China)	#BM3874
HRP Anti-rabbit secondary antibody	BOSTER Biotech (China)	#7074

**Critical commercial assays**		

Protein A Magnetic beads	Selleck (China)	#23202
Streptavidin Magnetic beads	Beyotime (China)	#P2150
Cell Counting Kit-8 (CCK-8)	DoJinDo (China)	#CK04
Transwell assay kit	Corning (USA)	#354480
Lipofectamine™ 3000	Invitrogen (USA)	#L3000-015
ECL substrate	Millipore (USA)	#WBKL S0100
SYBR Green Master Mix	Accurate Biotech (China)	#AG11701
Dual-Luciferase Reporter Assay System	Promega (USA)	#E1910
ChIP assay kit	Beyotime (China)	#P2078

**Experimental models: Cell lines**		

T47D	ATCC (USA)	#85102201
BT-549	ATCC (USA)	#HTB-122
MDA-MB-231	ATCC (USA)	#HTB-26
MCF-7	ATCC (USA)	#HTB-22
HEK293T	ATCC(USA)	RRID:CVCL_0063

**Chemicals, peptides, and recombinant proteins**		

MCP-MBP	Gu Lab^40^	
Reverse Transcription System	Accurate Biotech (China)	#AG11728
Recombinant RNase Inhibitor	Accurate Biotech (China)	#AG11608
TRIzol	TianGen (China)	#RK145

### Resource availability

#### Lead contact

Further information and requests for resources and reagents should be directed to the lead contact, Wei Gu (weigu1@yahoo.com).

#### Materials availability

Plasmids and cell lines generated in this study are available from the lead contact upon request.

### Experimental model and subject details

#### Cell lines

Primary MDA-MB-231, BT-549, T47D and MCF-7 cell lines were purchased from ATCC (the American type culture collection). Cells were cultured and passaged according to standard instructions of the ATCC. Cell lines were tested by determination of short-tandem repeats profiling through PCR following the instructions of the ATCC. The latest test was performed at October 2020.

#### Cell culture

Breast cancer cell lines and stable cell lines expressing MS2-tagged MACC1-AS1 and MACC1-AS1 truncates were grown in DMEM medium supplemented with 10% FBS, 100 units of penicillin/ml and 100 mg of streptomycin/ml, and were incubated at 37°C and 5% CO2 in a humidified chamber.

### Method details

#### PCR primers

PCR primers and chimeric antisense oligonucleotide (ASO) were purchased from IGE Biotech (Guangzhou, China). MACC1-AS1 siRNA and DDX5 siRNA were purchased from Gene Pharma (Suzhou, China) and are listed in Table S1.

#### Plasmid constructs

Human MACC1-AS1 cDNA was amplified by RT-PCR from MDA-MB-231 cells as previously described.^23^ Individual MACC1-AS1 truncates were PCR amplified and cloned into the pCIP2 lentivirus plasmid at the Not I and Bam HI sites. MS2-tagged MACC1-AS1 and its truncated variants were constructed by introducing a six-repeat MS2 hairpin structure into the BamHI site of a pCIP2 lentivirus plasmid. PsiCHECK 2 was purchased from Promega (USA) and used for luciferase reporter construction and luciferase assays. To construct the luciferase reporters, difference lengths of the *MACC1* promoter were PCR amplified and cloned into the 5’ end of the Renilla luciferase gene of the PsiCHECK 2 plasmid. Primer pairs used in the cloning experiments are described in Tables S1 and S2. All constructs were verified by sequencing analysis.

#### SgRNAs designing and cloning

CRISPR guide RNA (sgRNA) sequences were selected based on the criteria of having high on-target score^41^ and low off-target effects.^42^ Sequences of sgRNA#1, TTCATTTCGGGTACTGTCAAGGG, is located at -1840 bp to -1818 bp of the *MACC1* promoter and sgRNA#2: ATGAGGCCTTGCCTTCAAATAGG, located at -1549 bp to -1527 bp of the *MACC1* promoter. The sgRNAs were subcloned into a lentiviral plasmid pLC5-NC (IGE Biotech (GuangZhou, China), which contains a GFP marker. All the procedures for cloning of sgRNAs and preparing lentiviral particles for cell infection were commissioned manufacturing at IGE Biotech.

#### CRISPR genome editing procedure

The two vector approach was used to create CRISPR-induced knockout of the *MACC1* distal promoter in BT-549 cells.^43^ In this approach, a Cas9-expressing plasmid pCDH-CAG-CAS9-T2A-HygR was first transduced into the cells according to the manufacturer’s instruction. Following selection by HygR (400μg/ml) for 5 days, Cas9-expressing stable cells were collected and verified by RT-PCR. Cas9-expressing cells were then infected with sgRNA-expressing pLC5-NC lentiviral particles to generate knockout cells. 24-hours post transfection, cells were cultured in regular DEME medium. According to the green fluorescence intensities, stable cells with green color were sorted by FACS (fluorescence-activated cell sorting), then cultured and expanded by plating to progressively larger plates until there were enough cells to freeze and to use. Knockout efficiency of the *MACC1* distal promoter was verified by PCR and by Sanger seducing of the deleted genomic region of the *MACC1* gene.

#### RNA purification and qRT-PCR analysis

Total RNA from breast cancer cells was isolated with TRIzol (Invitrogen) following the manufacturer’s instructions. Concentration of isolated RNA was determined with a Nanodrop (Agilent). One microgram of total RNA was reverse transcribed into cDNA using a Reverse Transcription System (Tiangeng, China) according to the manufacturer’s protocol. Quantitative real-time PCR (RT-qPCR) was performed by SYBR Green Master Mix (Tiangen, China) using a Real Time PCR System (Applied Biosystems). Primers used in the PCR assay are described in Tables S1 and S2. To amplify primary MACC1 RNA, the primers were designed within the region between the fifth exon and the fifth intron. GAPDH mRNA was used as an internal control and for normalization. All experiments were replicated at least three times.

#### MS2-tagged RNA pulldown assays

Pulldown assays of MS2-tagged MACC1-AS1 were performed as previously described using a recombinant fusion protein (MBP-MCP) that contains a maltose-binding domain (MBP) and a domain (MCP) that recognizes the MS2 hairpins.^24^ Briefly, MBP-MCP-conjugated amylose resin (NEB, USA) was incubated with nuclear extracts prepared from cells expressing MS2-tagged MACC1-AS1 variants at 4°C for 4 h in the presence of RNase and protease inhibitors. After extensive washing, bound MACC1-AS1-MS2 RNP complexes were eluted with 100 μl lysis buffer containing 20 mM maltose. Aliquots of the eluted materials were used for analyzing precipitated MACC1-AS1, and the rest was used to detect its associated protein(s) by western blotting.

#### Luciferase assays

MDA-MB-231 or BT-549 cells were transfected with individual psiCHECK-2 constructs containing different region of the *MACC1* promoter using Lipofectamine® 3000 Reagent (Invitrogen, USA) according to the manufacturer’s instruction. After transfection for 36 h, the cells were harvested for Firefly/Renilla luciferase assays using the Dual-Luciferase Reporter Assay System (Promega, USA). Luciferase activities were normalized to the empty psiCHECK-2 plasmid.

#### Western blotting

Cells were cultured to confluence and washed with PBS. Cell extracts and cytoplasmic/nuclear fractions were prepared as previously described.^24^ Proteins were resolved by 4-12% SDS polyacrylamide gel electrophoresis and transferred to a nitrocellulose membrane. Blots were incubated with antibodies against DDX5 (1:1000), SP1 (1:1000), hnRNPD (1:1000) or GAPDH (1:2000 dilution) overnight at 4°C followed by incubation with HRP-conjugated secondary antibody (1:5000) for 1 hr at room temperature. Signals were detected using a ECL kit (Invitrogen, USA). For sequential immunoblotting experiments, the membranes were washed with Tris-buffered saline and treated with Western Blot Stripping Buffer (Thermo Scientific, USA) for 1 h. After washing and re-blocking, the membranes were incubated with other primary antibodies if necessary.

#### Bioinformatic studies

The potential relationship between MACC1-AS1 and MACC1 mRNA in human breast tumors was investigated using on line dataset, which comprised RNA-seq data from GEPIA database (https://gepia2.cancer-pku-cn). The two-gene correlation map is realized by the R software package. Spearman’s correlation analysis was used to describe the correlation between quantitative variables without a normal distribution. A p-value of less than 0.05 was considered statistically significant.

#### Cell transfections

Transfection of siRNAs or antisense oligonucleotides (ASO) for RNA knockdown were conducted by using Lipofectamine™ 3000 transfection reagent (Invitrogen, USA) as previously described.^24^^,^^44^

#### Chromatin isolation by RNA purification (ChIRP)

The ChIRP experiments were performed essentially using a protocol as previously described.^24^^,^^25^ Briefly, confluent cell culture expressing MS2-taged full-length MACC1-AS1 or truncates was washed in plates with PBS then treated with 1% formaldehyde for 10 min. Formaldehyde was quench with 125 mM glycine for 5 min. Cells were scrubbed from the plates and centrifuged at 3,000 rpm for 10 min. After carefully aspirate all liquid, cell pellets were resuspended in lysis buffer and sonicated for 5 min until lysate was clear. DNA was extracted from 5 μl aliquot and run on a 1 % agarose gel to make sure that the bulk DNA was around 500 bp. Sonicated cell lysates were centrifuged at 16,000 rcf for 10 min at 4°C and the supernatant was transferred to fresh tubes. To pulldown exogenous MACC1-AS1, amylose resin conjugated with recombinant MBP-MCP was incubated with the MACC1-AS1-MS2-containing supernatant at 4°C overnight in the presence of RNase and protease inhibitors. After extensive washing, MACC1-AS1-MS2 was eluted with 100 μl lysis buffer containing 20 mM maltose. Aliquots of the eluted material were used to extract RNA by TRIzol for analyzing enrichment of MACC1-AS1 RNAs by RT-PCR. The remaining material was used for isolating and analyzing associated DNAs by PCR. Where indicated, endogenous MACC1-AS1 was pulldown by using biotin-labeled oligonucleotides complementary to the 5’-end of MACC1-AS1 as previously described.^25^ Biotin-labeled probes were synthesized at RIBBIO Co (Shanghai, China) and the sequences were provided in Table S2.

#### Chromatin immunoprecipitation (ChIP)

Cells overexpressing MACC1-AS1 or truncated MACC1-AS1 variants were grown to more than 80% confluence in 10- or 15-cm plates. ChIP assays were performed using a kit from Beyotime Biotech (Shanghai, Chian) according to the manufacture’s instruction. Briefly, cells were crosslinked with 1% formaldehyde for 10 min followed by the addition of glycine to quench unreacted formaldehyde and washed three times with cold PBS. Cells were lysed and centrifuged in a microcentrifuge at 1000 rpm for 5 min in 4°C. After carefully removing supernatants, nuclear pellets were re-suspended in 0.5 ml of lysis buffer and sonicated to broken down chromatin into appropriate small fragments. Sonicated nuclear debris were removed by centrifugation. Supernatant was aliquoted and stored at -80°C freezer. For ChIP assays, anti-DDX5, anti-SP1 or normal IgG was mixed with nuclear extract and incubated at 4°C overnight with gentle rotation. The extracts were then incubated with protein A beads for 4 h at 4°C to capture DNA fragments associated with the proteins. The beads were extensively washed with buffers and the protein/DNA complexes were eluted and reverse cross-linked to free the proteins. Aliquots of solution were used for analyzing precipitated proteins by western blotting. The rest was used for DNA isolation and qPCR. Agarose gel electrophoresis was carried out to confirm the PCR products. Where indicated, the MMP-2 promoter was used as an internal control. Primers used in PCR or qPCR are listed in Table S1.

#### Cytoplasmic and nuclear fraction preparation

Nuclear and cytoplasmic extraction Kit was purchased from Absin Bioscience Inc (Shanghai, China). Briefly, cells were washed twice with PBS and resuspended in hypotonic buffer. NP40 was added to a final concentration of 0.5% and the cell suspension was vortexed for 10 s. After centrifugation at 3000 rpm for 10 min, the supernatant was collected as the cytosol fraction, from which cytoplasmic RNA was extracted. The pellet was resuspended and washed once with cold PBS. Pellet was collected as nuclear fraction from which RNA was isolated with Trizol.

### Quantification and statistical analysis

#### Statistical analysis

Statistical analysis was performed using SPSS 18.0 software and is presented as mean ± SEM. Statistical significance of results compared to control values was analyzed using Student’s t test (two tailed). A ∗*P* < 0.05 value indicates the experimental results to be statistically significant.

## Data Availability

• Data reported in this paper and additional information required to reanalyze the data reported in the study are available from the lead contact upon request. • This paper does not report any original code.
