# Supplementary material for: LncRNA MACC1-AS1 associates with DDX5 to modulate MACC1 transcription in breast cancer cells
Source: iScience. 2023 Aug 15;26(9):107642. doi: 10.1016/j.isci.2023.107642 (PMC10474461; doi:10.1016/j.isci.2023.107642)
Supplement: Document S1. Figures S1–S5 and Tables S1 and S2 [file mmc1.pdf]

iScience, Volume *26*

Supplemental information

LncRNA MACC1-AS1 associates with DDX5 to modulate *MACC1* transcription in breast cancer cells

Guiyu Zheng, Yanmei Zhu, Liqun Xu, Shaoying Chen, Xiaona Zhang, Wei Li, Weibin Chen, Yanchun Zhou, and Wei Gu

Suppl Fig. S1

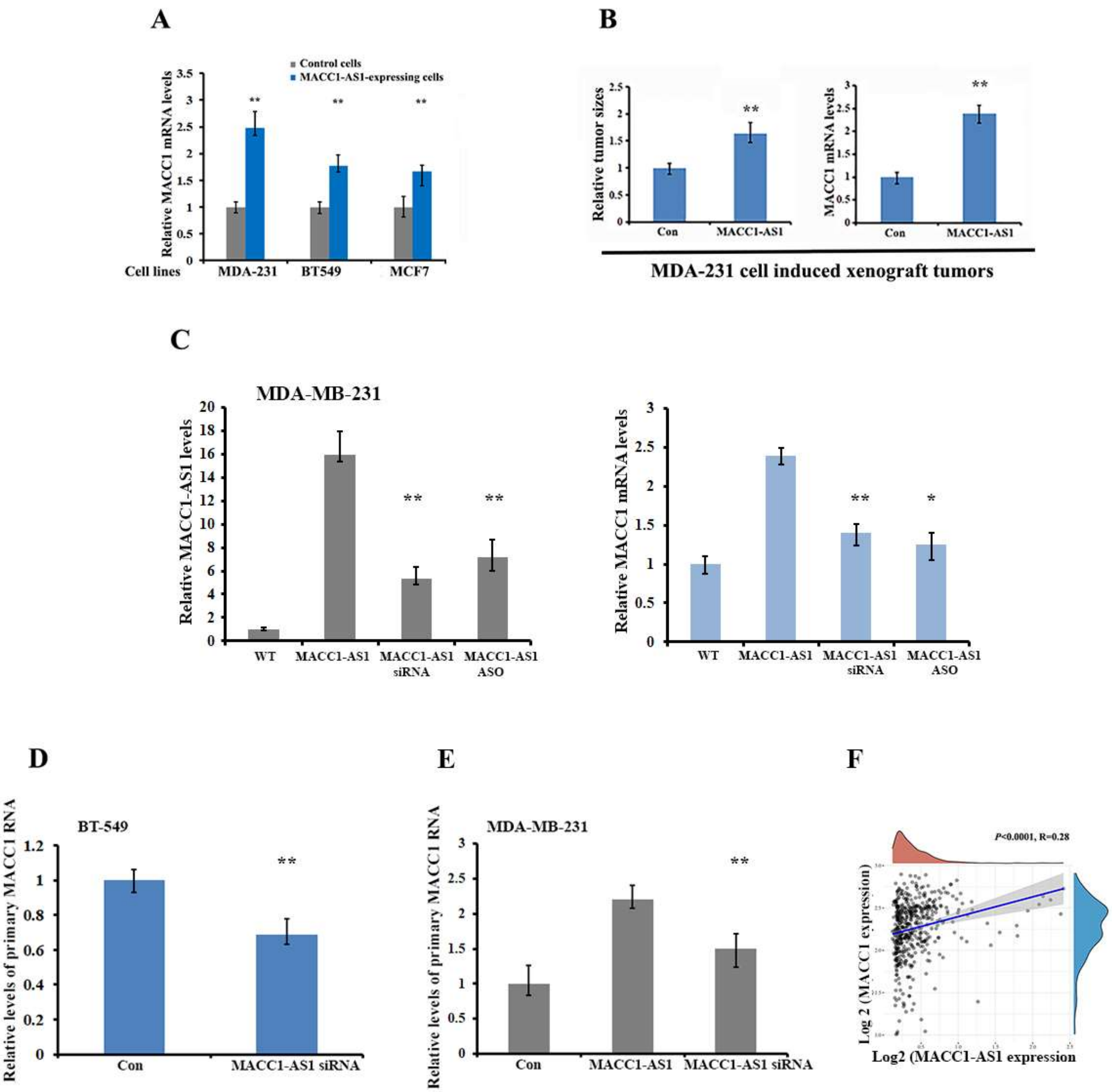

A
Control cells
MACC1-AS1-expressing cells
Relative MACC1 mRNA levels
Cell lines
MDA-231
BT549
MCF7
B
Relative tumor sizes
MACC1 mRNA levels
Con
MACC1-AS1
MDA-231 cell induced xenograft tumors
C
MDA-MB-231
Relative MACC1-AS1 levels
Relative MACC1 mRNA levels
WT
MACC1-AS1
MACC1-AS1 siRNA
MACC1-AS1 ASO
D
BT-549
Relative levels of primary MACC1 RNA
Con
MACC1-AS1 siRNA
E
MDA-MB-231
Relative levels of primary MACC1 RNA
Con
MACC1-AS1
MACC1-AS1 siRNA
F
P<0.0001, R=0.28
Log 2 (MACC1 expression)
Log2 (MACC1-AS1 expression

Suppl Fig. S2

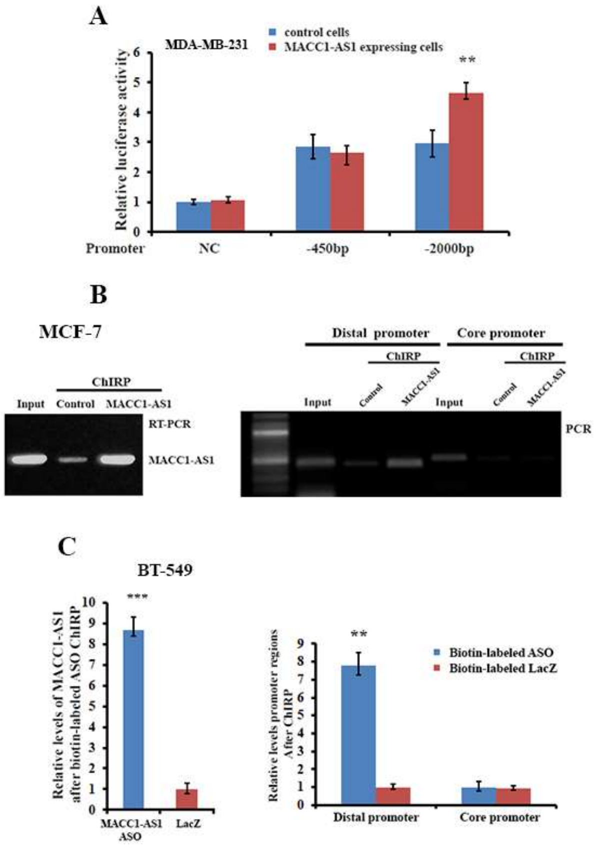
A
MDA-MB-231
control cells
MACC1-AS1 expressing cells
Relative luciferase activity
**
Promoter
NC
-450bp
-2000bp
B
MCF-7
ChIRP
Input
Control
MACC1-AS1
RT-PCR
MACC1-AS1
Distal promoter
Core promoter
ChIRP
Input
Control
MACC1-AS1
PCR
C
BT-549
***
Relative levels of MACC1-AS1 after biotin-labeled ASO ChIRP
MACC1-AS1 ASO
LacZ
**
Biotin-labeled ASO
Biotin-labeled LacZ
Relative levels promoter regions After ChIRP
Distal promoter
Core promoter

Suppl Fig. S2D

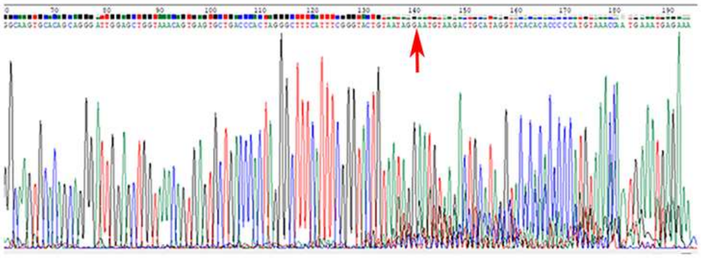

Sequence alignment of MACC1 promoter

```
BT-WT          1 acatgggactgaggactctagggagcccagtccttggggagctgttagaaaggcaagtgc
BT-KO          1 acatgggactgaggactctagggagcccagtccttggggagctgttagaaaggcaagtgc

BT-WT         61 acagcagggattggagctggtaaacagtgagtgctgacccactagggctttcatttcggg
BT-KO         61 acagcagggattggagctggtaaacagtgagtgctgacccactagggctttcatttcggg

BT-WT        121 tactgtcaagggttcaattgctagatttaatgctttgcctttctgcaaataaaggctgtg
BT-KO        121 tactgt------------------------------------------------------

BT-WT        181 ggagccacaccagaccagactgcaggggattctgataacagtactcatgcatggcattt
BT-KO            ------------------------------------------------------------

BT-WT        241 aggtagctttttaaatgtaaatgttttccgggagacataaagatggcattaggcattaac
BT-KO            ------------------------------------------------------------

BT-WT        301 acacacacaccttagagttaacatctcagttattttatgtgttgattgtttatcacaaac
BT-KO            ------------------------------------------------------------

BT-WT        361 atctacttgtacattaatgagttaatgaagtagggatacaatgaggccttgccttcaaat
BT-KO        127 ---------------------------------------------------------aat

BT-WT        421 aggaatgtaagactgcataggtacacacacccccatgta
BT-KO        130 aggaatgtaagactgcataggtacacacacccccatgta
```

Suppl Fig, S3

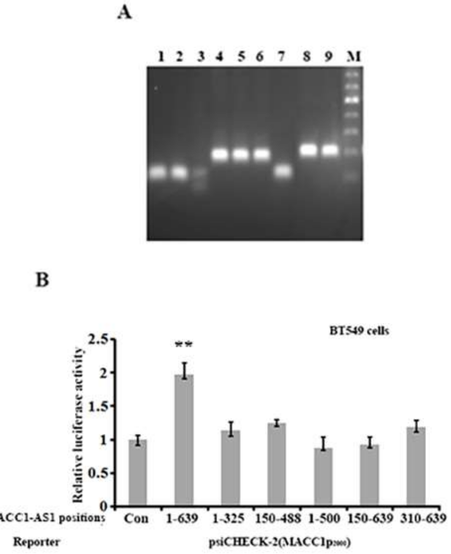
A
1 2 3 4 5 6 7 8 9 M
B
BT549 cells
Relative luciferase activity
2.5
2
1.5
1
0.5
0
**
MACC1-AS1 positions
Con
1-639
1-325
150-488
1-500
150-639
310-639
Reporter
psiCHECK-2(MACC1p2000)

Suppl. Fig. S4

A

In vitro purified proteins associated with MACC1-AS1

Protein name	Mass(Da)	Area in vitro MACC1-AS1-MS2	-10lgP	Peptides	Unique
PTBP1	57221	1.46E+06	188.18	7	7
DDX5	69148	3.95E+05	148.07	7	5
HNRNPL	64133	3.07E+05	192.87	6	6
HNRNPD	38434	5.96E+05	184.3	6	5
HNRNPAB	36225	2.98E+05	164.44	5	5
HNRNPUL2	85105	3.45E+05	146.02	6	6
MYBBP1A	148854	6.44E+04	145.64	4	4

B

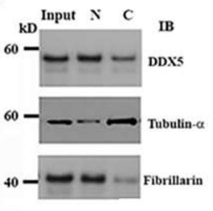

C

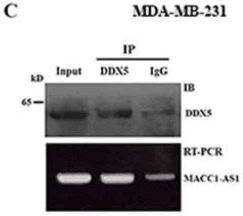

D

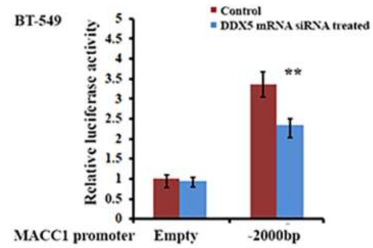

Suppl Fig. S5

A

-500

```
TTTGAGTGACAATCACAGTGCTGATGTAGAGGGAAAGGGGGAACTAGTTAGACACTGTCA
                                  SP-1                  c-Jun
CTCACCTGGGAAGGCTTTATTCACCTGTTCCACAGGGCAGTGAGGCACCTTCAGCTCTGA
ATCACCGAAAGAGAATCTGGTGGGGCAAGTTCCAGCTGCATGAGGATTTGCTTGCATAAA
TATTTTTTACTTATTGCTAACACTGAGGGTGCCTTCTTACTCCCTGGCAAACATTAAACC
ACTTTTATTTCCTTTCATGGAAATAAGATTATATTTACAGATGGTTCTTAGATATACTCA
CCTGATTTTTTTTTAATTGCTTTTCCACCTGCTTCCCCTTTCTTCTTAGGGTGAAACTCT
AGCCATACGCCCTCTTCTGGTTTCGGGTGAGGAGCCTGAATTGTGGGTATCGTAGTTCTC
TTGCCTTTTGGGGTTTCTAGTTGGGCAGCTTTGGAGCCACAGTGGTAGAACTTCAAGTCC
CAGGTGGCTCAGGAAGCAGGGGTGCAGTTGCCTGCCTGCATACGGAGCAAGGCATGTTTG
                                                            -1
```

B

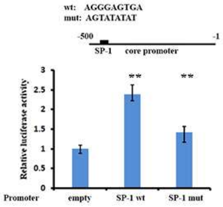

C

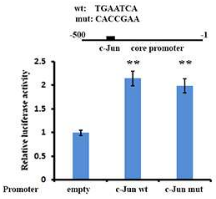

D

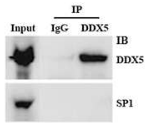

Table S1

Primers	Nucleotide Sequences
SacI-MACC1p -2000-F	gagctcGGAGCTGATAATCAAAAATC
SacI-MACC1p -1500-F	gagctcACCCATGTAAACCAATGAAA
SacI-MACC1p -1000-F	gagctcTTTTTCTTTGTCACCATTCTG
SacI-MACC1p -450-F	gagctcGAAGGCTTTATTCACCTGTTC
NheI-MACC1p -1-R	gctagcGCAGGCAGGCAACTGCACCC
NheI-MACC1p -550-R	gctagcCCCAGGTGAGTGACAGTGTC
MACC1p-mut-Sp1-F	TTCACCTGTTCCACAGTATATATAGGCACCTTCAGCTCTG
MACC1p-mut-Sp1-R	CAGAGCTGAAGGTGCCTATATATACTGTGGAACAGGTGAA
(RT-PCR) MACC1-F	CATTTTCGGTCAGGAAGAATTGC
(RT-PCR) MACC1-R	TGGAAGCATTATTACCACGAAGG
MACC1p-dis -806F	GATTTAATGCTTTGCCTTTCTGC
MACC1p-dis -1476R	CTCATTTCATTGGTTTACATGGG
MACC1p-core -450F	GAAGGCTTTATTCACCTGTTC
MACC1p-core -18R	GCAGGCAGGCAACTGCACCC
MACC1-exo5-F	TGGGTTAGAAGCAGACAGTTGA
MACC1-int5-R	GATTGTAACTCACAGTGCCACCT
MMP-2p For	CAAGAGTGAGTGGGGAATTCGT
MMP-2p Rev	AACAGTATGCAGTGAAGAAGCC
GAPDH-F	GAGTCAACGGATTTGGTCGT
GAPDH-R	TGGGATTTCCATTGATGACA

Table S2

**Primers**	**Nucleotide Sequences**
MACC1-AS1-1F	CTCGAGCAAATTGTAGAATACACACACAC
MACC1-AS1-639R	GCGGCCGCAACTGGGAGTCAATTTTTATTGAA
MACC1-AS1-488R	CTCGAGAGATGACTTGGTGAGAGCAGAAG
MACC1-AS1-125F	TCTAGAGGCAAGGTTGCCTTCTTAATGAT
MACC1-AS1-325R	CTCGAGCACCATTTAATCAATGCAGATCT
MACC1-AS1-(qPCR1-F)	TTATTTCATGTGTTCTATCCTCAG
MACC1-AS1-(qPCR1-R)	CTTCTATTCACAACCTGTTCCTC
MACC1-AS1-(qPCR2-F)	AATGGTGACTACAAATAGACTTA
MACC1-AS1-(qPCR2-R)	ATGACTTGGTGAGAGCAGA
Chimeric MACC1-AS1 ASO	mGmUmGmUmGTGTGTGCAGTmGmUmGmUmG
Biotin-labeled MACC1-AS1 Probe for ChIRP	AGATGACTTGGTGAGAGCAG-/3bio/
Biotin-labeled LacZ probe for ChIRP control	GCCAGTGAATCCGTAATCAT-/3bio/

Supplemental information

Figure S1 Correlative expression of MACC1-AS1 and MACC1 in breast cancer cells, related to Figure 1

(A) Levels of MACC1 mRNA were detected by qRT-PCR in three breast cancer cell lines following stable expression of MACC1-AS1. (B) Left, MACC1-AS1 facilitated growth of mouse xenograft breast tumors. Right, MACC1-AS1 up-regulated MACC1 mRNA expression in xenograft tumors. (C) Levels of MACC1-AS1 and MACC1 mRNA were examined by qRT-PCR in MACC1-AS1-expressing MDA-MD-231 cells, in which MACC1-AS1 was silenced by siRNA or ASO (antisense oligonucleotide). *P < 0.1, **P < 0.01. (D) and (E) Levels of primary MACC1 RNA were measured by qRT-PCR, using the primers located in the fifth exon and adjacent fifth intro, in BT-549 cells where MACC1-AS1 was silenced by siRNA and in MACC1-AS1 expressing MDA-MB-231 cells. **P < 0.01. (F) Spearman correlation analysis of the relationship between MACC1-AS1 and MACC1 mRNA expression using comprised mRNA-seq data from GEPIA tumor database. The horizontal axis in the figure represents the expression distribution of MACC1-AS1, and the vertical axis is the expression distribution of MACC1 mRNA. The top side values represent the correlative p value and the correlation coefficients (R).

Figure S2 MACC1-AS1 interacts with the distal promoter of the MACC1 gene, related to Figure 2

(A) luciferase reporters driven by the -450 bp or -2000 bp of the MACC1 promoter were transfected into MDA-MB-231 cells with or without MACC1-AS1 expression. Luciferase activities were determined after 36 h transfection using a dual-luciferase assay system. Renilla luciferase activity was normalized to the activity of firefly luciferase. **P < 0.01 as determined by Student's t test. (B) ChIRP experiments were performed to detect interaction of MACC1-AS1 with MACC1 distal promoter in MCF-7 cells expressing MS2-tagged MACC1-AS1. Left: Enrichment of MACC1-AS1 in the ChIRP precipitates were measured by RT-PCR. Right: PCR showed that MACC1-AS1 was preferentially interacted with the distal promoter of the MACC1 gene. (C) ChIRP experiments using biotin-labeled antisense oligonucleotide (ASO) were performed to pulldown endogenous MACC1-AS1 and its associated MACC1 promoter region in BT-549 cells. Left panel: MACC1-AS1 levels in the ChIRP precipitates were analyzed by RT-qPCR. Right panel: qPCR indicated that the distal region of the MACC1 promoter was preferentially interacted with MACC1-AS1. (D) Sanger sequencing of the MACC1 promoter indicated that the distal region of the MACC1 gene was deleted in BT-549 KO cells. Upper, the red arrowhead points to the region where the distal promoter was deleted. Lower, sequence comparison of the distal promoters of WT and the KO cells.

Figure S3 The full-length of MACC1-AS1 is required for MACC1 promoter activity, related to Figure 3

(A) MDA-MB-231 cell lines stably expressing MS2-tagged full-length or truncated MACC1-AS1 was established. Relative expressions of MACC1-AS1 variants were verified by RT-PCR. DNA bands in each lane indicates the corresponding MACC1-AS1 expressed in the tested cell lines. Lanes 1 and 2: 1-639 (full-length); lane 3: control cells; lane 4: (1-500); lane 5: (150-639); lane 6: (310-639); lane 7: (150-488) and lanes 8 and 9: (GAPDH control); M: DNA ladders. (B) Luciferase reporter driven by the MACC1p2000 and vector expressing full-length or truncated MACC1-AS1 were co-transfected into BT-549 breast cancer cells. Luciferase activity was determined after 36 hr incubation. Data is presented as means±SD from three independent experiments. **P<0.01 as determined by Student's t-test.

Figure S4 DDX5 associates with MACC1-AS1, related to Figure 4

(A) In vitro pull-down assay was performed using synthesized MACC1-AS1-MS2 and total cell lysates 42. Proteins co-precipitated with MACC1-AS1 were analyzed by MS spectrometric assays and were partially listed. DDX5 is one of the proteins to associate with MACC1-AS1. (B) Cytoplasmic and nuclear fractions of MACC1-AS1-expressing cells were prepared. Quality of the fractions was tested by western blots using tubulin and fibrillarin antibodies. (C) Upper: western blots of IP experiments using DDX5 antibody and the nuclear lysates of BT-549 cells. Lower: RT-PCR demonstrated that MACC1-AS1 was precipitated with DDX5. (D) BT-549 cells with or without DDX5 mRNA siRNA treatment were transfected with the luciferase reporters driven by the -2000 bp MACC1 promoter. Luciferase activities were determined after 36 h transfection using a dual-luciferase assay system. Renilla luciferase activity was normalized to the activity of firefly luciferase. **P < 0.01.

Figure S5 Recruitment of SP-1 to the MACC1 core promoter activates transcription, related to Figure 6

(A) A 500 bp MACC1 basal promoter sequence was shown. The sequence spans the nucleotides -500 to -1 upstream of the MACC1 gene, which harbors the binding sites for SP-1 shown in red color and for c-Jun in blue color. (B) and (C) Upper: a schematic representation of a luciferase reporter driven by the -500 bp core promoter of the MACC1, in which the SP-1 or the c-Jun binding site was mutated, respectively. Lower: luciferase activity was determined after transfecting the reporter into MDA-MB-231 cells. Data is presented from three independent experiments. **P<0.01. (D) Immunoprecipitation assays were performed using DDX5 and normal IgG antibodies in MDA-MB-231 cells. DDX5 and SP-1 do not interact with each other.

Table S1 Primers used for generating MACC1 promoter and luciferase constructs, related to STAR Methods

Red base pairs indicate the nucleotide sequences of restriction sites for construct cloning. Underlined base pairs represent the nucleotide sequences which have been mutated to generate mutant MACC1 promoter fragments.

Table S2 Primers used for generating MACC1-AS1 constructs and qRT-PCR, related to STAR Methods.

Red base pairs indicate the restriction sites for cloning of MACC1-AS1 constructs.
